# Evaluating the Efficacy of Ropivacaine Instillation Through a Subgaleal Drain for Postcraniotomy Pain: A Randomized Controlled Trial

**DOI:** 10.7759/cureus.92892

**Published:** 2025-09-22

**Authors:** Usha Shukla, Urvashi Yadav, Deepika Doneria, Priyanka Bhardwaj

**Affiliations:** 1 Anesthesiology and Critical Care, Uttar Pradesh University of Medical Sciences, Saifai, IND; 2 Anesthesiology, Uttar Pradesh University of Medical Sciences, Saifai, IND

**Keywords:** brain drain, craniotomy, drug instillation, postcraniotomy pain, ropivacaine

## Abstract

Background: The majority of patients experience moderate to severe postcraniotomy pain, which causes several complications such as raised intracranial pressure, cerebral hemorrhage, chronic headache, etc. Therefore, this study aimed to assess the efficacy of ropivacaine instillation through subgaleal drain in patients undergoing elective craniotomy to reduce postcraniotomy pain. The primary objective of this study was to assess postoperative pain after craniotomy using the Numeric Rating Scale (NRS) score.

Materials and methods: A prospective, double-blinded, randomized controlled study was conducted in 120 patients posted for elective craniotomy, randomly allocated into three groups. Group A (n = 40) received 12 mL of 0.9% normal saline, Group B (n = 40) received 12 mL of 0.1% ropivacaine, and Group C (n = 40) received 12 mL of 0.2% ropivacaine. Postoperative NRS score, duration of analgesia, amount of rescue analgesia, patient satisfaction score, and hemodynamic parameters were compared in 24 hours among the three groups.

Results: The NRS score was lowest in Group C compared to Group A and Group B at all time points, with significant differences observed at 30 minutes, 45 minutes, one hour, and two hours after drug instillation. The duration of analgesia was significantly prolonged in Group C (489.24 ± 204.5 minutes) compared to Group B (112.7 ± 156.5 minutes) and shortest in Group A (43.87 ± 24.2 minutes). The amount of rescue analgesic required was least in the 0.2% ropivacaine group (103.12 ± 36.3 mg) compared to the 0.1% ropivacaine group (144.37 ± 48.5 mg). It was in a substantial amount in the 0.9% normal saline group (176.2 ± 61.7 mg).

Conclusions: We concluded that for postcraniotomy pain, a single-dose 0.2% ropivacaine drug installation at the end of surgery through a surgical (subgaleal) drain had better pain control, a longer duration of analgesia, and a lower analgesic requirement compared to 0.1% ropivacaine and 0.9% normal saline.

## Introduction

Craniotomy is a common neurosurgical procedure in which a part of the skull is removed temporarily to expose the brain for performing intracranial procedures [1]. Moderate to severe postcraniotomy pain has often been reported, which causes several complications such as raised intracranial pressure, cerebral hemorrhage, brain herniation, and even chronic headache. Therefore, adequate pain relief is an important aspect of neuroanesthesia [2,3]. Several interventions have been tried over a period in the postcraniotomy pain management to improve outcomes and patient satisfaction, such as preoperative gabapentin administration, which decreases anesthetic and analgesic consumption up to 48 hours after surgery. Still, it also delays tracheal extubation and increases sedation postoperatively [4]. Intraoperative administration of various drugs like opioids (e.g., remifentanil), IV acetaminophen, dexmedetomidine, ketamine, corticosteroids, and NSAIDs reduces postoperative complications by reducing postcraniotomy pain, but they have their side effects [5-7]. Scalp infiltration with local anesthetics has several advantages, including decreased hemodynamic response to the placement of head fixation devices and surgical incisions. Still, it is effective in the reduction of pain scores after craniotomy in the first few hours only, not for the long term, and it does not reduce the need for postoperative pain medications [8]. To overcome this limitation, ropivacaine instillation through the subgaleal drain concept has been developed, which significantly shows a reduction in pain scores after craniotomy, and it also reduces the need for postoperative pain medications [9]. We aimed to compare the efficacy of two concentrations (0.1% and 0.2%) of ropivacaine in reducing postcraniotomy pain. The primary objective of this study was to assess postcraniotomy pain using the Numeric Rating Scale. The secondary objectives were to compare the duration of analgesia, the rescue analgesic requirement in 24 hours postoperatively, the postoperative hemodynamic parameters, and the patient satisfaction scores.

## Materials and methods

This prospective, randomized, controlled, double-blind study was conducted in the Department of Anesthesiology, Uttar Pradesh University of Medical Sciences, Saifai, India, from December 2023 to December 2024, following approval from the institution's ethical committee with approval number 65/2023-24 and with CTRI no. CTRI/2023/12/060896. Informed and written consent was taken after explaining the procedure and its related risks from all 120 patients. Patients of either sex, aged 18-60 years, with American Society of Anesthesiologists Physical Status (ASA-PS) grade I and II, posted for elective craniotomy at the U.P. University of Medical Sciences and Research, Saifai, were considered for the study. Patients who were not giving consent, had previous head trauma, had a history of craniotomy, had chronic headache, had a Glasgow Coma Scale (GCS) <13, had active psychiatric disorders, had an allergy to the study drug, or had chronic opioid and steroid use were excluded from the study.

Sample size was calculated based on a previous study conducted by Sonal et al. [10]; the mean differences in Numeric Rating Scale (NRS) scores at 24 hours in the normal saline versus ropivacaine 0.1% group were 0.58 ± 0.76, in the normal saline versus ropivacaine 0.2% group 1.06 ± 1, and in the ropivacaine 0.1% versus ropivacaine 0.2% group 0.48 ± 0.76. Using the power of study 80%, confidence interval 95%, and an alpha error of 0.05, the minimum required sample size was calculated to be 40.

A total of 120 patients were assessed for eligibility and included in the study with no exclusions. Randomization was done by using a computer-generated random number table to ensure baseline comparability between the groups. Allocation concealment was maintained by using sequentially numbered, opaque sealed envelopes, ensuring that the assignment of 40 participants to each group was unbiased and unaffected by the enrolling personnel. Three groups were allocated: Group A (control group) received 12 mL of 0.9% normal saline (n = 40), Group B (study group) received 12 mL of 0.1% ropivacaine (n = 40), and Group C (study group) received 12 mL of 0.2% ropivacaine (n = 40).

This is a double-blind study; the patients enrolled in the study were blinded to the concentration of ropivacaine instilled, and the staff personnel who prepared the study drug were not involved in the study. The anesthesiologist who administered the drug and the observer who recorded all data were also blinded to the used drug concentration. With informed consent, all patients were explained how to rate the intensity of pain using NRS [11], a scale of 0-10, where 0 = no pain, 5 = moderate pain, and 10 = worst pain, and the patient satisfaction score [12], which was indicated as 1 = extremely dissatisfied, 2 = dissatisfied, 3 = somewhat dissatisfied, 4 = undecided, 5 = somewhat satisfied, 6 = satisfied, and 7 = extremely satisfied, to assess the patient satisfaction. All patients were instructed to fast according to the recent NPO guidelines on the day before surgery [13]. An IV line was secured with an 18-G cannula. All patients received a tablet of alprazolam 0.5 mg at night before surgery and an injection. Ranitidine 150 mg IV half an hour before surgery. The baseline parameters (heart rate (HR), blood pressure, and oxygen saturation) were taken. The NRS score and GCS were assessed before shifting the patient to the operating theater.

In the operating room, ASA standard monitors, including electrocardiography, peripheral oxygen saturation (SpO2), and non-invasive blood pressure, were connected to the patients. All the patients were premedicated with 10 mcg/kg glycopyrrolate, 0.05 mg/kg midazolam, and 2 mcg/kg fentanyl and preoxygenated for three minutes. Induction was done with 2 mg/kg propofol IV, followed by muscle relaxation with 0.1 mg/kg vecuronium IV. Intubation was performed with an appropriately sized cuffed endotracheal tube. Adequate anesthesia was maintained with 33% oxygen, 66% nitrous oxide, and 0.6-1.0% isoflurane. In this study, we avoided lignocaine scalp infiltration. The scalp was infiltrated with an appropriate concentration of adrenaline with normal saline in all patients. Adequate muscle relaxation was ensured by intermittent injections of 0.02 mg/kg vecuronium and a paracetamol injection of 1 g/100 mL for intraoperative pain, administered 30 minutes after intubation. IV fentanyl 1 mcg/kg, along with 4 mg of ondansetron, was given at the time of dural closure. Once a subgaleal drain was placed and subcutaneous tissue sutured, the patients received the study drug according to the group allocation, and the exact time was noted. The study drug was instilled through a subgaleal drain, which was then clamped for 15 minutes to avoid leakage. After the instillation of the drug, skin closure and wound dressing were performed. At the end of surgery, muscle relaxation was reversed with neostigmine 0.05 mg/kg IV and glycopyrrolate 10 mcg/kg. The patient was then extubated after generating an adequate tidal volume.

In the postoperative room, the vital parameters like HR, systolic blood pressure (SBP), diastolic blood pressure (DBP), mean arterial pressure (MAP), SpO2, and postcraniotomy surgical site pain score were assessed at 15-minute intervals in the first hour, then every two hours up to six hours, and every six hours up to 24 hours. Pain was assessed as per the NRS score, and if at any time NRS > 4 was observed, IV diclofenac 75 mg was given as a primary analgesic. The time for the first analgesic required was recorded in minutes as the "duration of analgesia." The total number of doses of analgesics given in 24 hours was recorded. Adverse effects like postoperative nausea and vomiting were also recorded. Patient satisfaction score was measured at 12 hours and 24 hours after surgery.

Statistical analysis

**Figure 1:**
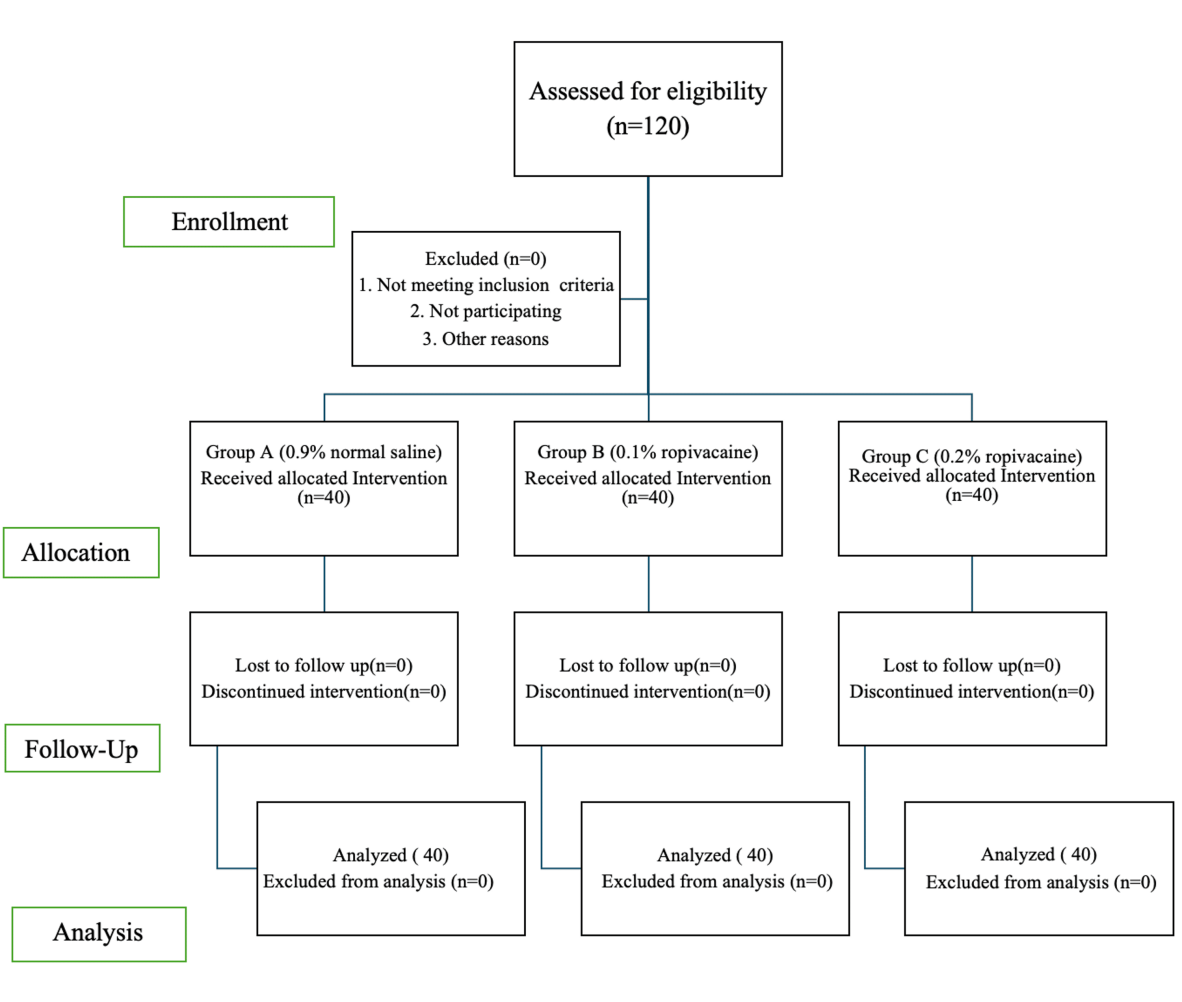
CONSORT diagram CONSORT: Consolidated Standards of Reporting Trials

All data were recorded, summarized, tabulated, and statistically analyzed using SPSS Statistics version 24.0 (IBM Corp. Released 2016. IBM SPSS Statistics for Windows, Version 24.0. Armonk, NY: IBM Corp.). The statistical presentation and analysis of this study were conducted, and the results are presented as mean ± SD and percentages. The chi-square test was used to compare the categorical variables among the three groups. An unpaired t-test was used for intergroup comparison. The one-way analysis of variance was used to compare the continuous variables among the groups at different periods. The p-value < 0.05 was considered significant. A total of 120 patients were recruited for the study, and none were excluded, as shown in the Consolidated Standards of Reporting Trials (CONSORT) flow diagram (Figure 1).

## Results

All three groups were comparable in terms of their demographic data (age, gender, weight, height, body mass index, and ASA grade) (Table 1).

**Table 1:** Comparison of demographic profile of patients among the three groups ^1^ANOVA test, ^2^Chi square test, Group A: 0.9% normal saline group, Group B: 0.1% ropivacaine group, Group C: 0.2% ropivacaine group, BMI: body mass index, n: number of patients, ASA: American Society of Anesthesiologists, SD: standard deviation, M: male, F: female, p > 0.05: statistically nonsignificant

Variables	Group A (n = 40) (mean ± SD)	Group B (n = 40) (mean ± SD)	Group C (n = 40) (mean ± SD)	Test values
Age (years)	43.35 ± 15.37	45.23 ± 17.06	48.23 ± 14.35	P = 0.374, F^1 ^= 0.991
Gender (M: F)	20:20	22:18	20:20	P = 0.875, X^2 ^= 0.267
Weight (kg)	65.20 ± 6.04	65.10 ± 6.61	67.20 ± 8.77	P = 0.519, F^1 ^= 0.660
Height (cm)	159.17 ± 8.66	160.03 ± 9.13	160.30 ± 8.38	P = 0.562, F^1 ^= 0.579
BMI (kg/m^2^)	25.73 ± 1.23	25.42 ± 1.64	26.07 ± 1.97	P = 0.169, F^1 ^= 2.185
ASA (class I/II)	21/19	21/19	16/24	P = 0.434, X^2 ^= 1.669

**Figure 2:**
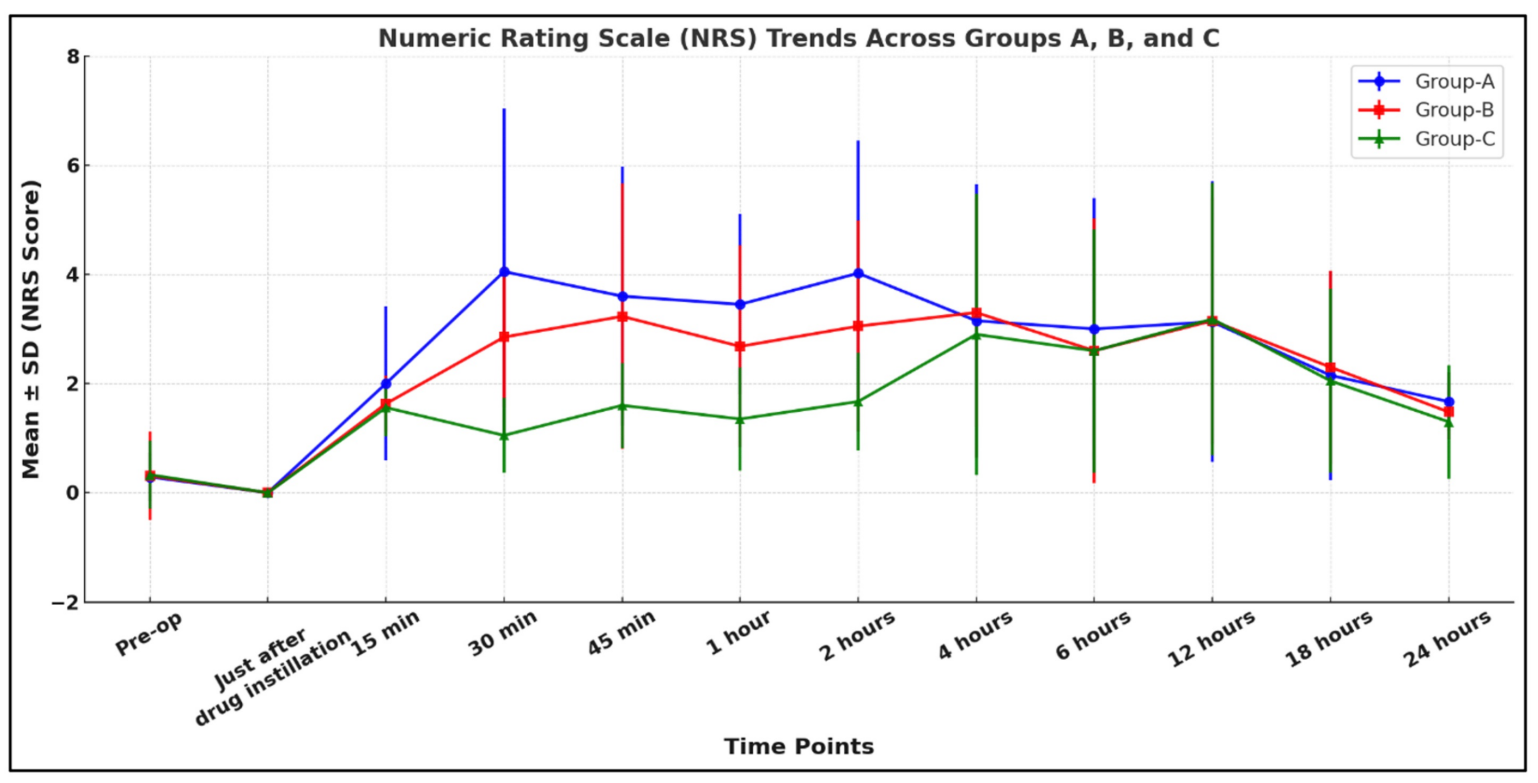
Comparison of postoperative NRS score among the study groups NRS: Numeric Rating Scale, SD: standard deviation, Group A: 0.9% normal saline group, Group B: 0.1% ropivacaine group, Group C: 0.2% ropivacaine group

Figure 2 shows that the comparison of NRS scores among Groups A, B, and C over time revealed statistically significant differences at various intervals. At 15 minutes, Group A had a slightly higher pain score (2.00 ± 1.41) compared to Group B (1.63 ± 0.52) and Group C (1.56 ± 0.53), with an overall significant difference (p = 0.018) and a significant difference between Group A and C (p = 0.040). The most pronounced differences occurred at 30 minutes, with Group A (4.05 ± 2.98) showing the highest score compared to Group B (2.85 ± 1.29) and Group C (1.05 ± 0.68), all comparisons being highly significant (overall p < 0.0001). This trend continued for 45 minutes and 1-2 hours, during which Group C consistently showed the lowest pain scores (all p < 0.001). From four to 24 hours, differences in pain scores diminished, and p-values indicated no significant differences.

The duration of analgesia was significantly prolonged in the 0.2% ropivacaine group (489.24 ± 204.5 minutes) compared to the 0.1% ropivacaine group (112.7 ± 156.5 minutes) and was the shortest in the 0.9% normal saline group (43.87 ± 24.2 minutes). The amount of rescue analgesic required was least in the 0.2% ropivacaine group (103.12 ± 36.3 mg) compared to the 0.1% ropivacaine group (144.37 ± 48.5 mg). It was in a substantial amount in the 0.9% normal saline group (176.2 ± 61.7 mg) (Table 2).

**Table 2:** Duration of analgesia and analgesic requirement among the study groups ^#^ANOVA test, **unpaired t-test, *p < 0.05: statistically significant, SD: standard deviation, Group A: 0.9% normal saline group, Group B: 0.1% ropivacaine group, Group C: 0.2% ropivacaine group

	Group A (mean ± SD) n = 40	Group B (mean ± SD) n = 40	Group C (mean ± SD) n = 40	P-value^#^ (overall)	A vs. B		B vs. C		A vs. C	
Duration of analgesia (min)	43.87 ± 2.27	112.73 ± 156.55	489.24 ± 204.54	F = 135.64 *p < 0.0001	*p = 0.048	^**^t = 2.02	*p < 0.0001	^**^t = 9.53	*p < 0.0001	^**^t = 10.48
Amount of rescue analgesic (mg)	176.25 ± 61.73	144.37 ± 48.57	103.12 ± 36.30	F = 23.85 *p < 0.0001	*p = 0.013	^**^t = 2.58	*p < 0.0001	^**^t = 4.28	*p < 0.0001	^**^t = 6.00

**Figure 3:**
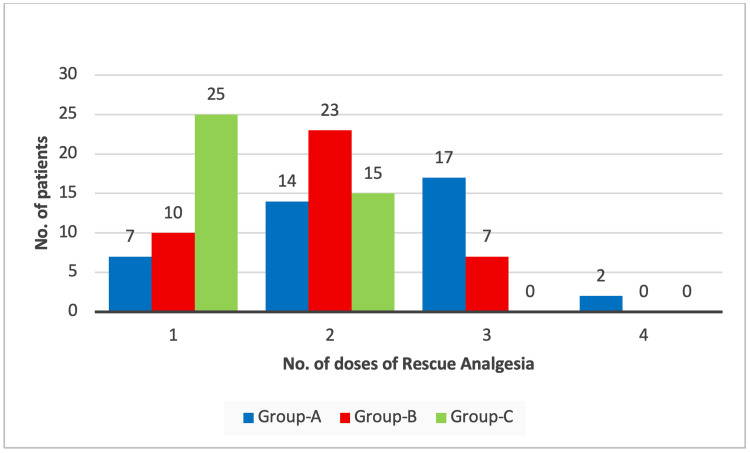
Comparison of number of doses of rescue analgesia received among the study groups Group A: 0.9% normal saline group, Group B: 0.1% ropivacaine group, Group C: 0.2% ropivacaine group

Figure 3 shows that the number of demands for rescue analgesics required was significantly different among the groups (chi-square = 38.343, p < 0.0001*). A greater number of patients in Group C required only 1 dose of rescue analgesia compared to Groups A and B.

Patient satisfaction score was significantly higher in the 0.2% ropivacaine group (6.40 ± 0.49) and (6.20 ± 0.83) at 12 hours and 24 hours, respectively, compared to the 0.1% ropivacaine group (4.37 ± 1.2), (4.60 ± 1.19), and was lowest in the 0.9% normal saline group (2.12 ± 0.9), (2.35 ± 1.12) at 12 hours and 24 hours, respectively (Table 3).

**Table 3:** Comparison of patient satisfaction score among the study groups ^#^ANOVA test, ** unpaired t-test, *p < 0.05: statistically significant, SD: standard deviation, Group A: 0.9% normal saline group, Group B: 0.1% ropivacaine group, Group C: 0.2% ropivacaine group

Time	Group A (mean ± SD) n = 40	Group B (mean ± SD) n = 40	Group C (mean ± SD) n = 40	p-value^#^ (overall)	A vs. B		B vs. C		A vs. C	
12 hours	1.70 ± 0.48	4.10 ± 1.85	6.50 ± 0.53	F = 395.12 ^*^p < 0.0001	^*^p < 0.0001	^**^t = 7.47	^*^p < 0.0001	^**^t=6.34	^*^p < 0.0001	^**^t = 38.29
24 hours	1.70 ± 0.48	4.70 ± 1.49	6.70 ± 0.48	F = 601.25 ^*^p < 0.0001	^*^p < 0.0001	^**^t = 9.97	^*^p < 0.0001	^**^t=6.96	^*^p < 0.0001	^**^t = 43.64

**Figure 4:**
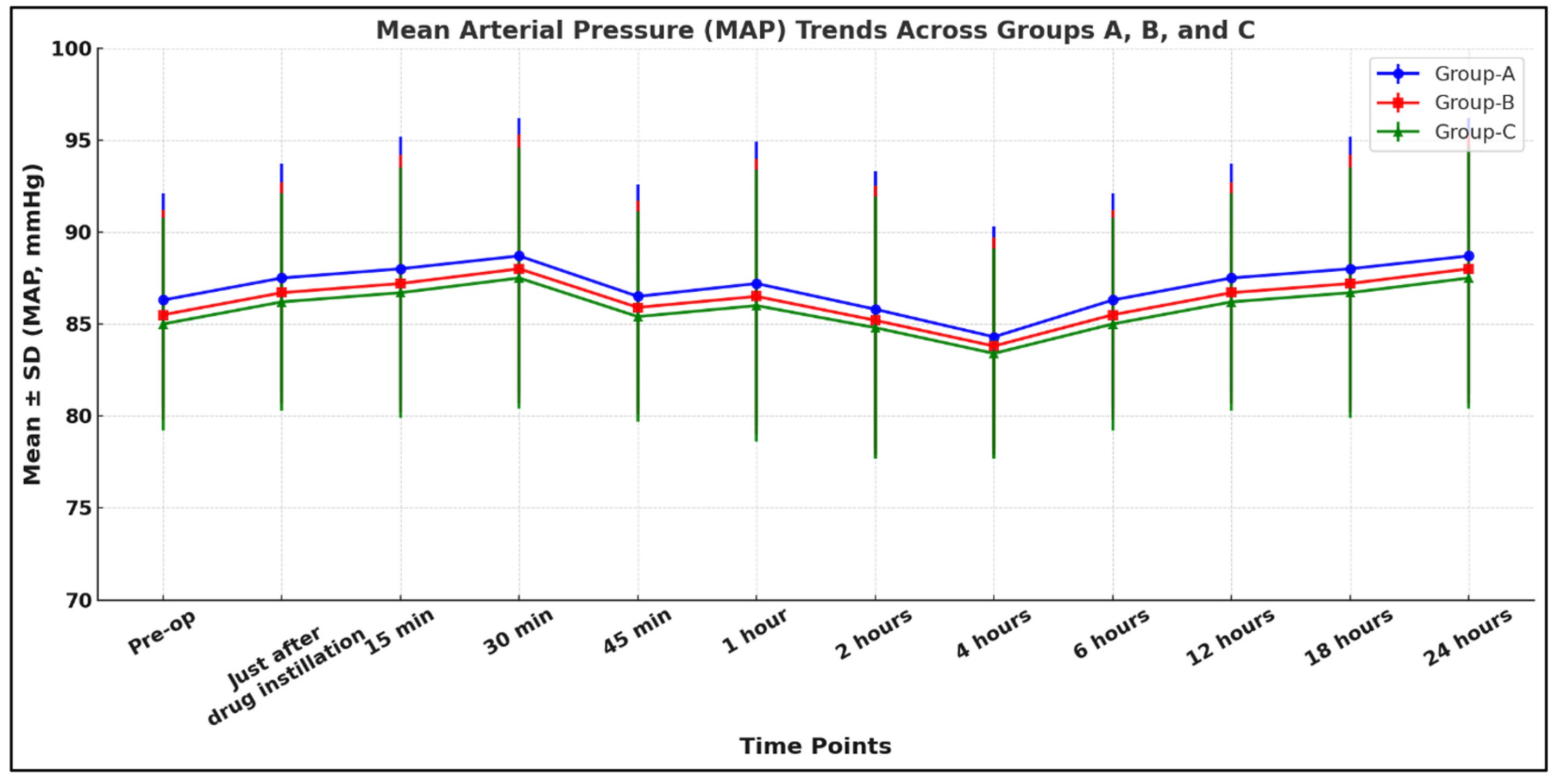
Comparison of MAP among the study groups Group A: 0.9% normal saline group, Group B: 0.1% ropivacaine group, Group C: 0.2% ropivacaine group, MAP: mean arterial pressure, SD: standard deviation

**Figure 5:**
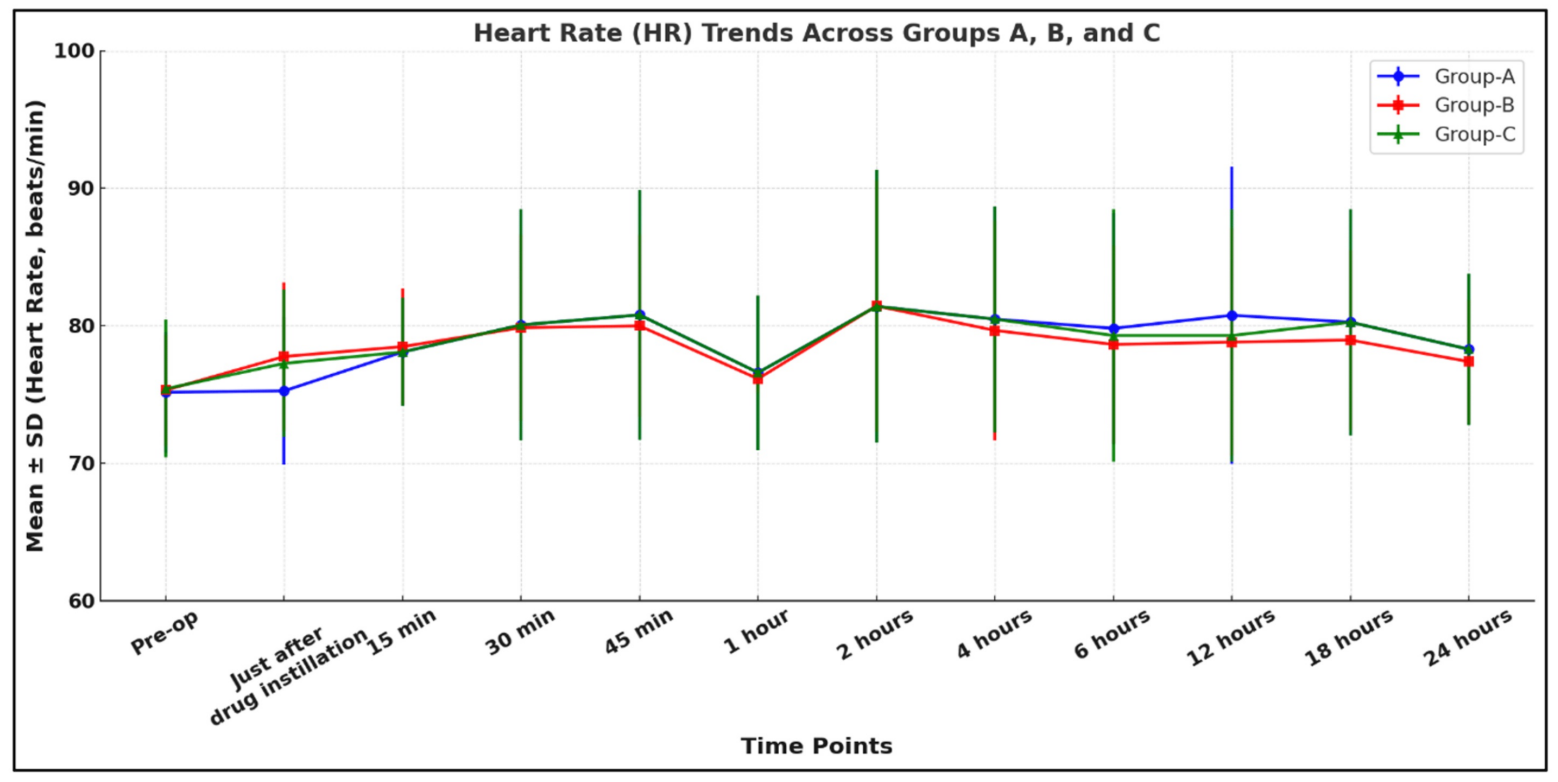
Comparison of HR among the study groups Group A: 0.9% normal saline group, Group B: 0.1% ropivacaine group, Group C: 0.2% ropivacaine group, HR: heart rate, SD: standard deviation

**Figure 6:**
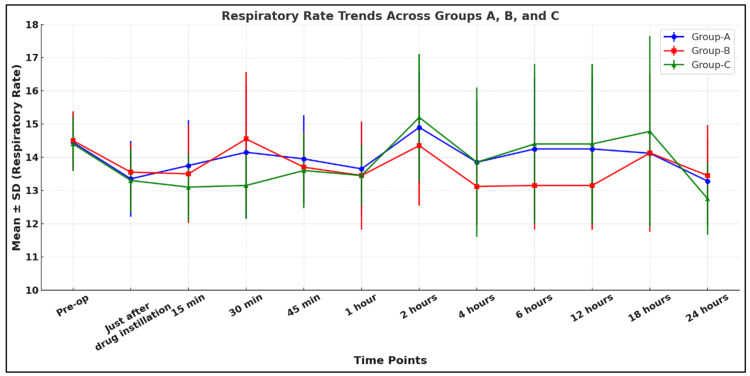
Comparison of RR among the study groups Group A: 0.9% normal saline group, Group B: 0.1% ropivacaine group, Group C: 0.2% ropivacaine group, RR: respiratory rate, SD: standard deviation

Our study shows that all three groups were comparable in terms of hemodynamic parameters like HR, MAP, and respiratory rate (RR) (p > 0.05) (Figures 4-6).

## Discussion

In the present study, we compared the effects of two concentrations of ropivacaine (0.1% and 0.2%) with the control group, 0.9% saline, through subgaleal drain in relieving the postcraniotomy pain and associated complications with different parameters such as NRS scores, duration of analgesia, amount of rescue analgesic required, hemodynamic parameters, and patient satisfaction score.

In our study, patients who received 0.2% ropivacaine showed the lowest mean values of NRS score at all points of time as compared to patients who received 0.1% ropivacaine and 0.9% normal saline. In an intergroup comparison, a significant difference (p < 0.05) was found between Groups A and C and between Groups B and C at 30 minutes, 45 minutes, one hour, and two hours. Therefore, the 0.2% ropivacaine group showed better outcomes in terms of postoperative analgesia for the first few hours. In favor of our study, Saini and Yadav found that the NRS score was lower in the 0.25% ropivacaine group (20 mL) at all time intervals compared with the 0.9% normal saline group (20 mL) [14]. Similarly, Bala et al. found that the pain score, which was assessed by VAS score in their study, was higher in the normal saline group (12 mL) than in the 0.25% ropivacaine (12 mL) at all time points [9]. Li et al. also observed that the VAS score postoperatively was lower in the ropivacaine group (3.2 ± 1.4) than in the control group (4.0 ± 1.4) [15], aligning with our results.

The duration of analgesia was significantly prolonged in Group C (489.24 ± 204.54 minutes) compared to Group B (112.725 ± 156.55 minutes) and shortest in Group A (43.87 ± 24.27 minutes). Saini and Yadav also found that the duration of analgesia was significantly (p < 0.05) longer in the ropivacaine group (12.15 ± 1.49) compared to the normal saline group (4.3 ± 1.03) [14]. Similarly, Bala et al. found that the mean value of duration of analgesia (in minutes) was less in the normal saline group (108.20 ± 102.80) than in the ropivacaine group (480.0 ± 276.8) [9]. Deng et al. compared the safety and efficacy of three ropivacaine concentrations (0.2%, 0.3%, and 0.4%) for pectoral nerve block in patients undergoing modified radical mastectomy with those who did not receive the block. Additionally, they found that the duration of analgesia was longer in the ropivacaine groups than in the control group [16].

During intergroup comparison, the amount of rescue analgesic required was least in Group C (103 ± 36.3 mg) compared to Group B (144.3 ± 48.5 mg). In a substantial amount in Group A (176.2 ± 61.7 mg), and this difference was found to be statistically significant (p < 0.05). Saini and Yadav similarly found that the total amount of rescue analgesic requirement was much higher in the normal saline group (191.25 ± 38.28) than the ropivacaine group (97.5 ± 35.2) [14]. Consistently, Swennen et al. studied the effect of intraoperative local infiltration analgesia with ropivacaine (group R) on postoperative pain for patients undergoing thoracolumbar junction fracture surgery and compared it with the control group, which did not receive the ropivacaine (group M). They reported that morphine consumption was lower (5.6 ± 6.3) in group R and higher (10.8 ± 6.6) in group M [17].

The number of demands for rescue analgesics required was significantly different among the groups (p < 0.05). A greater number of patients in Group C required only one dose of rescue analgesia compared to Groups A and B. Saini and Yadav found similar results:12 patients in group N required three doses of analgesic. No patient in group R required analgesic doses, which indicates that group R is better than group N [14]. Likewise, Bala et al. found that a greater number of analgesic doses were consumed in the normal saline group (2.48 ± 0.59) than the ropivacaine group (1.12 ± 0.44) [9].

Our study showed that all three groups were comparable in terms of hemodynamic parameters like HR, SBP, DBP, MAP, SpO2, and RR (p > 0.05). Bala et al. found similar, nonsignificant changes in SBP, DBP, and MAP (p > 0.05), which is consistent with our study [9]. Yang et al. conducted a study to compare the effect of scalp nerve block with ropivacaine (0.2%, 0.3%, 0.5%) on postoperative pain in patients undergoing craniotomy with the control group (normal saline). They also found nonsignificant changes postoperatively in HR and MAP (p > 0.05), which supported our study [18].

The patient satisfaction score was significantly higher in Group C both at 12 hours (6.40 ± 0.49) and 24 hours (6.20 ± 0.83) compared to Group B (4.37 ± 1.25) and (4.60 ± 1.19), respectively, and was lowest in Group A (2.125 ± 0.91) and (2.35 ± 1.12), respectively. Schug et al. [19] investigate the dose-response relationship of extradural ropivacaine infusion. All the patients were divided into four groups: ropivacaine 0.1%, 0.2%, 0.3%, or saline at a rate of 10 mL/hr for 21 hours. Patient satisfaction was higher in the 0.2% and 0.3% ropivacaine groups than in the two other groups. These results were in concordance with our study, in which higher ropivacaine concentrations (0.2%) show better satisfaction scores than 0.1% ropivacaine and normal saline.

Some patients in our study showed postoperative nausea and vomiting, but the difference was not statistically significant among the groups (p > 0.05). None of our patients showed adverse events related to local anesthetic toxicity.

Limitations

The sample size was small, which may limit the generalizability and statistical power of our findings. Further studies are required with a large sample size to clinically extrapolate the results. We have studied only the first 24 hours of the postoperative period, so the results should be confirmed in a study with longer follow-up. Plasma concentrations of ropivacaine were not measured in our study. Further studies can be planned in the future by measuring plasma concentrations also. It will give us an insight into the absorption characteristics of ropivacaine when given through the drain and its local effect, in addition to its effect after systemic absorption.

## Conclusions

From this study, it can be concluded that subgaleal instillation of 0.2% ropivacaine significantly reduces postcraniotomy pain in the first two hours, prolongs the duration of analgesia, decreases the requirement for rescue analgesics, and improves patient satisfaction compared to 0.1% ropivacaine and normal saline. The technique was safe, with no significant adverse effects observed, and maintained stable hemodynamic parameters. These findings support the use of 0.2% ropivacaine through subgaleal drain as an effective and simple strategy for improving postoperative pain management in patients undergoing elective craniotomy. Further studies with larger sample sizes and longer follow-up periods are warranted to validate these results and explore the pharmacokinetics of ropivacaine via this route.
